# Characterization of dysfunctional breathing using cardiopulmonary exercise testing

**DOI:** 10.14814/phy2.70388

**Published:** 2025-06-02

**Authors:** Sebastian F. Möbus, Chris J. Harding, Catherine L. Taylor, Karl P. Sylvester, Jonathan P. Fuld

**Affiliations:** ^1^ School of Clinical Medicine University of Cambridge Cambridge UK; ^2^ Lung Function Unit Cambridge University Hospitals NHSFT Cambridge UK; ^3^ Sports and Exercise Medicine East Suffolk and North Essex NHSFT Ipswich UK; ^4^ Respiratory Physiology Royal Papworth Hospital NHSFT Cambridge UK; ^5^ Victor Phillip Dahdaleh Heart and Lung Research Institute University of Cambridge Cambridge UK

**Keywords:** breathing pattern disorder, cardiopulmonary exercise testing, dysfunctional breathing, dyspnoea, exercise capacity, hyperventilation

## Abstract

Cardiopulmonary exercise testing (CPET) is emerging as a useful tool in the identification of dysfunctional breathing (DB). We aimed to evaluate the prevalence and functional impact of different patterns of DB in 628 adult patients referred for CPET due to unexplained dyspnoea (August 2019–December 2023). Patients were assigned to four groups following CPET interpretation: normal, breathing pattern disorder (BPD), hyperventilation (HV), and combined BPD with HV (BPDHV). Demographic and CPET performance data were analyzed using non‐parametric tests as appropriate. 94 (15.0%) patients had normal CPETs and 267 (42.5%) were identified as having DB. The remaining 267 were excluded as having alternative diagnoses. Of those with DB, 145 (54.3%) had BPD, 41 (15.4%) had HV, and 81 (30.3%) had BPDHV. VE/VCO_2_ was significantly increased in HV or BPDHV only (*p* < 0.001). Patients in all three DB groups exhibited significantly impaired peak VO_2_ compared to those with normal CPETs (*p* < 0.001). These CPET findings highlight DB as a common driver of symptoms in unexplained dyspnoea. Over half of patients with DB had isolated BPD, which requires visual inspection of relevant CPET plots to diagnose. Those identified with DB had significantly reduced peak VO_2_, which may be a useful classifier of functional severity in DB.

## 1 INTRODUCTION

Dysfunctional breathing (DB) encompasses a spectrum of conditions in which the normal biomechanical pattern of breathing is disrupted, resulting in dyspnoea disproportionate to any underlying disease pathology and significant impact on patients' quality of life (Boulding et al., 2016; Ionescu et al., 2020). Despite DB affecting up to 9.5% of the population (Thomas et al., 2005), the condition remains poorly characterized, with no gold standard diagnostic or classification system (Boulding et al., 2016; Ionescu et al., 2020; Ruane et al., 2024).

Traditionally, approaches to DB diagnosis have involved questionnaires such as the Nijmegen questionnaire (van Dixhoorn & Duivenvoorden, 1985). Such questionnaires rely on subjective reporting of symptoms and may only be designed to identify hyperventilation syndrome (van Dixhoorn & Folgering, 2015). Alternatively, physiotherapists may diagnose DB through protocols such as the Breathing pattern assessment tool (Todd et al., 2018); however, this requires specialized training and may not capture exercise‐related DB or allow quantification of DB severity. In addition, these questionnaires and tools do not shed light on whether any DB present is related to underlying pathophysiology. Cardiopulmonary exercise testing (CPET) is emerging as a useful tool in the objective diagnosis and characterization of DB patterns, with the additional benefit of ruling out potential underlying pathologies (Neder et al., 2018).

CPET provides quantitative data and visualized plots detailing the ventilatory, cardiac, gas exchange, and muscle responses to exercise (Albouaini et al., 2007; Mezzani, 2017). Comparison to normal predicted responses facilitates identification of pathological causes for exertional breathlessness, including DB (Neder et al., 2018). CPET is therefore well placed to rule out cardiorespiratory aetiologies for unexplained dyspnoea and potentially rule in DB (Bansal et al., 2018). Specific parameters and panels within the CPET nine‐panel plot can point to specific elements of DB, enabling detailed characterization of abnormal breathing responses (Ionescu et al., 2020). Furthermore, CPET includes indicators of exercise performance and therefore can provide an indicator as to the severity of functional impairment imposed on a patient by DB.

Previous attempts to classify DB have subdivided DB into thoracic and extra‐thoracic subtypes (Barker & Everard, 2015), with further division of thoracic DB based on the pathophysiological pattern of disruption to respiratory muscle activation (Boulding et al., 2016). Hyperventilation is by far the most studied subtype of DB; however, periodic deep sighing and other erratic breathing patterns are increasingly recognized and identifiable by CPET (Genecand et al., 2023; Knöpfel et al., 2024). In this study, we propose a simple classification system for DB according to patterns identifiable during CPET: breathing pattern disorder (BPD) (characterized by erratic or inappropriate changes in breathing frequency and/or tidal volume) and/or hyperventilation (HV).

This study therefore aimed to: (1) evaluate the prevalence of different patterns of DB in a population referred for CPET testing due to unexplained breathlessness, and (2) assess the functional impact of different patterns of DB using CPET indicators of exercise performance.

## 2 METHODS

This study retrospectively analyzed data from 704 adult patients referred to a tertiary centre (Addenbrooke's hospital) for CPET between August 2019 and December 2023. Criteria for exclusion were as follows: age <18, referral reason other than unexplained dyspnoea, referral for pre‐operative assessment, repeat or annual CPETs, and participants invited as part of research studies.

A standardized ramp‐incremental CPET protocol was used as detailed in the European Respiratory Society's statement on standardization of CPET testing in chronic lung diseases (Radtke et al., 2019). Prior to exercise, patient's baseline spirometry and observations were obtained. Spirometry was performed according to ERS/ATS guidance (Graham et al., 2019). CPETs were conducted using a cycle ergometer and a Vyntus CPX metabolic cart (Vyaire Medical, UK) with a resting, 3‐min unloaded, incremental, and recovery phase. Individualized work rate ramps calculated according to Cooper and Storer (2001) were used to target an incremental exercise phase of 8–12 min in duration. CPET data was collected using an 8‐breath rolling average. Demographic data for each patient was also obtained. Both physiological maximal CPETs and symptom‐limited submaximal tests were included in order to reflect the maximal functional capacity of the patients. Individual CPET data was interpreted using the Study of Health in Pomerania (SHIP) reference ranges for those 18 years and over (Gläser et al., 2013). Due to the SHIP values starting from an age of 25, for those patients aged 18–24 years, their age was manually modified in the system to 25 so that the SHIP values could be utilized.

**FIGURE 1:**
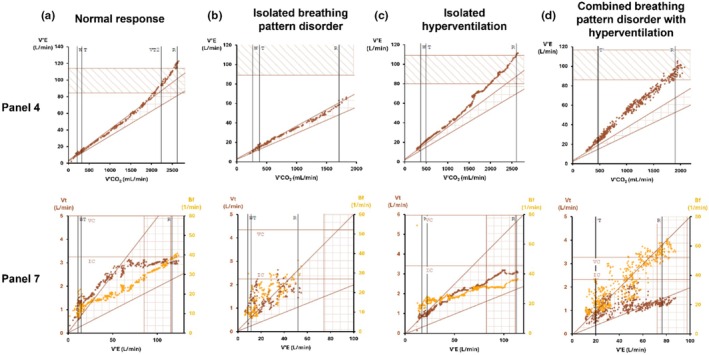
Examples of the utility of panel 4 and 7 in the Wasserman 9 panel‐plot in distinguishing between normal and different dysfunctional breathing responses to increasing exercise. Panel 4 plots VCO_2_ against minute ventilation (VE) allowing interpretation of ventilatory efficiency (VE/VCO_2_ slope). Panel 7 plots both breathing frequency (Bf) and tidal volume (Vt) against increasing minute ventilation, enabling visualization of exercise breathing responses. (a) Normal response – Normal VE/VCO_2_ slope and appropriate increases in Vt and Bf. (b) Isolated BPD – Normal VE/VCO_2_ slope but highly erratic changes in Vt and Bf. (c) Isolated HV – Raised VE/VCO_2_ slope but appropriate increase in Vt and Bf (d) Combined BPDHV – Raised VE/VCO_2_ slope and highly erratic changes in Vt and Bf.

Each CPET was physiologically interpreted by a chief respiratory physiologist and subsequently clinically interpreted by a consultant healthcare scientist and a respiratory consultant, utilizing the Wasserman 9 panel‐plot (Wasserman et al., 1987) plus an array of cardiovascular, ventilatory, and gas exchange variables acquired during CPET tests. Identification of patterns of DB via CPET involves careful interpretation of relevant panels and ventilatory parameters including the slope of the relationship between minute ventilation and carbon dioxide production (VE/VCO_2_), the ventilatory equivalents for carbon dioxide (VeqCO_2_) and end tidal CO_2_ (P_ET_CO_2_). The VE/VCO_2_ slope was calculated as the slope of the regression line prior to the respiratory compensation point (RCP) in order to exclude disproportionate increases in VE/VCO_2_ reflecting excessive metabolic acidosis in heavy exercise (Phillips et al., 2020). VeqCO_2_ and P_ET_CO_2_ are plotted continuously and their value at anaerobic threshold determined. Specific breathing pattern anomalies are identified utilizing the relationship between minute ventilation and both breathing frequency (B_f_) and tidal volume (V_t_). Example panels demonstrating normal and dysfunctional breathing responses are presented in Figure 1. CPET diagnosis of DB is also described extensively elsewhere (Ionescu et al., 2020; Neder et al., 2018). Following joint clinical and physiology review, four study groups of interest were identified as follows:

1. “Normal” – CPETs in which no pathological or nonpathological cause for unexplained dyspnoea was identified
2. “Isolated breathing pattern disorder (BPD)” – Patients in which erratic or inappropriate increases in tidal volume and/or breathing frequency were identified as a significant driver of dyspnoea without evidence of hyperventilation
3. “Isolated hyperventilation (HV)” – Patients in which isolated acute and/or chronic hyperventilation was identified as significant driver of dyspnoea without evidence of a BPD
4. “Combined breathing pattern disorder with hyperventilation (BPDHV)” – Patients in which both hyperventilation and breathing pattern disorders were identified as contributors to their symptoms

Patients in which alternative pathophysiology to the aforementioned study groups of interest was identified were excluded from further analysis.

Demographic data (Age, Sex, Height, Weight, and BMI) and CPET indicators of exercise performance including peak VO_2_ (mL/min/kg) and ventilatory efficiency (VE/VCO_2_) were analyzed and compared between subgroups. Patients' peak VO_2_ was further classified according to the Weber classification of functional impairment (Weber et al., 1982) (Table 1) and the distribution compared between subgroups. Finally, the relationship between ventilatory efficiency and peak VO_2_ was investigated.

**TABLE 1:** Weber classification of functional impairment.

Disease severity	Weber class	
		Peak VO_2_ (mL O_2_ • kg^−1^ • min^−1^)
Mild to none	A	>20
Mild to moderate	B	16–20
Moderate to severe	C	10–16
Severe	D	<10

*Note*: The exercise impairment of patients, as measured by their peak VO_2_ obtained during CPET testing, was classified as group A–D according to the Weber classification.

Ethical approval for the study was obtained from the EHR Research and Innovation (ERIN) Database Data Access Committee (CUH R&D Reference: A096993). The approving committee waived the requirement for informed consent for this retrospective analysis of de‐identified data.

### 2.1 Statistical analysis

Continuous data are presented as median (Inter‐quartile range). Intergroup analysis of demographic data and variables acquired during cardiopulmonary exercise testing was carried out using non‐parametric Kruskal–Wallis one‐way analysis of variance. Where appropriate, post hoc Dwass‐Steel‐Critchlow‐Fligner (DSCF) tests were performed to identify significant pairwise comparisons. Gender distribution was compared using a chi‐square test. The relationship between VE/VCO_2_ and peak VO_2_ was investigated using Spearman's rank correlation coefficient. Statistical analyses were performed using the Jamovi 2.3.21.0 package. Statistical significance was defined as *p* < 0.05 in all cases.

## 3 RESULTS

### 3.1 Prevalence of dysfunctional breathing within the referral population

**FIGURE 2:**
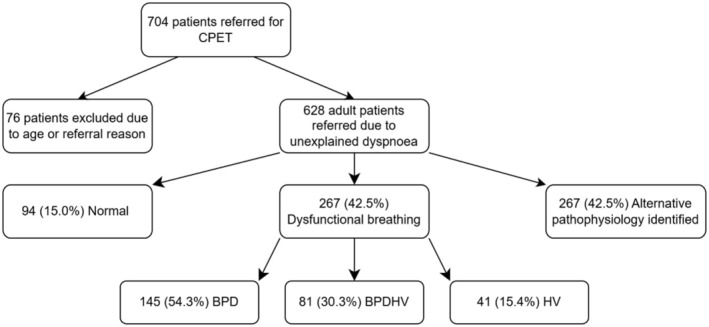
Flowchart depicting the identification of the four study groups and the prevalence of dysfunctional breathing within the referral population.

Between August 2019 and December 2023, 704 patients were referred for CPET, of whom 628 were adult patients referred for CPET investigation due to unexplained dyspnoea (Figure 2). Of these, 94 (14.9%) patients had normal CPETs, and 267 (42.4%) were clinically identified as having dysfunctional breathing. 267 patients were excluded due to the identification of alternative pathophysiology to DB. Of those with dysfunctional breathing, 145 (54.3%) had BPD, 41 (15.4%) had HV, and 81 (30.3%) had BPDHV.

### 3.2 Demographic data

Demographic data (Sex, Age, Weight, Height, and BMI) for each study group along with significant pairwise comparisons are presented in Table 2. Patients identified with DB were typically in their 50s or 60s and clinically overweight (BMI 25–29.9 kg/m^2^), with a slight female predominance. No significant difference in gender distribution between study groups was present (*p* > 0.05). Patients with isolated HV were significantly older than patients with isolated BPD (*p* < 0.001) or normal CPETs (*p* = 0.004), whilst patients with BPD were significantly younger than patients with BPDHV (*p* = 0.034). Patients with isolated BPD were significantly heavier than the normal group (*p* = 0.006). Height did not differ across the 4 groups (*p* > 0.05). Patients with isolated BPD or HV had significantly higher BMI compared with the normal group (*p* = 0.003 and *p* = 0.029, respectively).

**TABLE 2:** Comparison of demographic and CPET data between the four study groups.

	Study group				One‐way ANOVA
		Dysfunctional breathing			
	Normal	BPD	HV	BPDHV	*p*‐Value
*n*	94	145	41	81	
Sex (% Female)	60.60%	54.50%	53.70%	71.60%	0.070^†^
Age	53.0 (40.5–60.0)^b^	49.0 (35.0–59.0)^de^	61.0 (51.0–71.0)^bd^	53 (43.0–65.0)^e^	<0.001
Weight (kg)	72.5 (61.9–85.2)^a^	82.6 (67.8–95.0)^a^	80.4 (66.7–90.4)	74.7 (66.9–88.2)	0.011
Height (m)	1.69 (1.64–1.74)	1.70 (1.63–1.76)	1.68 (1.61–1.73)	1.66 (1.62–1.74)	0.184
BMI	25.2 (22.0–28.4)^ab^	27.5 (23.9–32.4)^a^	28.4 (25.1–31.7)^b^	27.4 (23.6–29.9)	0.002
VE/VCO_2_	28.0 (25.8–30.6)^bc^	29.5 (26.5–32.5)^de^	42.3 (34.7–47.8)^bd^	37.7 (32.3–46.2)^ce^	<0.001
Peak VO_2_ (mL/min/kg)	27.2 (21.9–33.0)^abc^	22.1 (17.6–26.7)^ade^	15.8 (12.1–21.0)^bd^	17.3 (14.0–21.8)^ce^	<0.001

*Note*: Data are presented as Median (Inter‐quartile range). The *p*‐values of Kruskal–Wallis one‐way analysis of variance tests are presented. Significant post‐hoc Dwass‐Steel‐Critchlow‐Fligner pairwise comparisons are indicated as follows: ^a^Normal vs. BPD, ^b^Normal vs. HV, ^c^Normal vs. BPDHV, ^d^BPD vs. HV and ^e^BPD vs. BPDHV. Significance was defined as *p* < 0.05.

^†^ Gender distributions were compared using a Chi‐Square test.

### 3.3 Ventilatory efficiency

**FIGURE 3:**
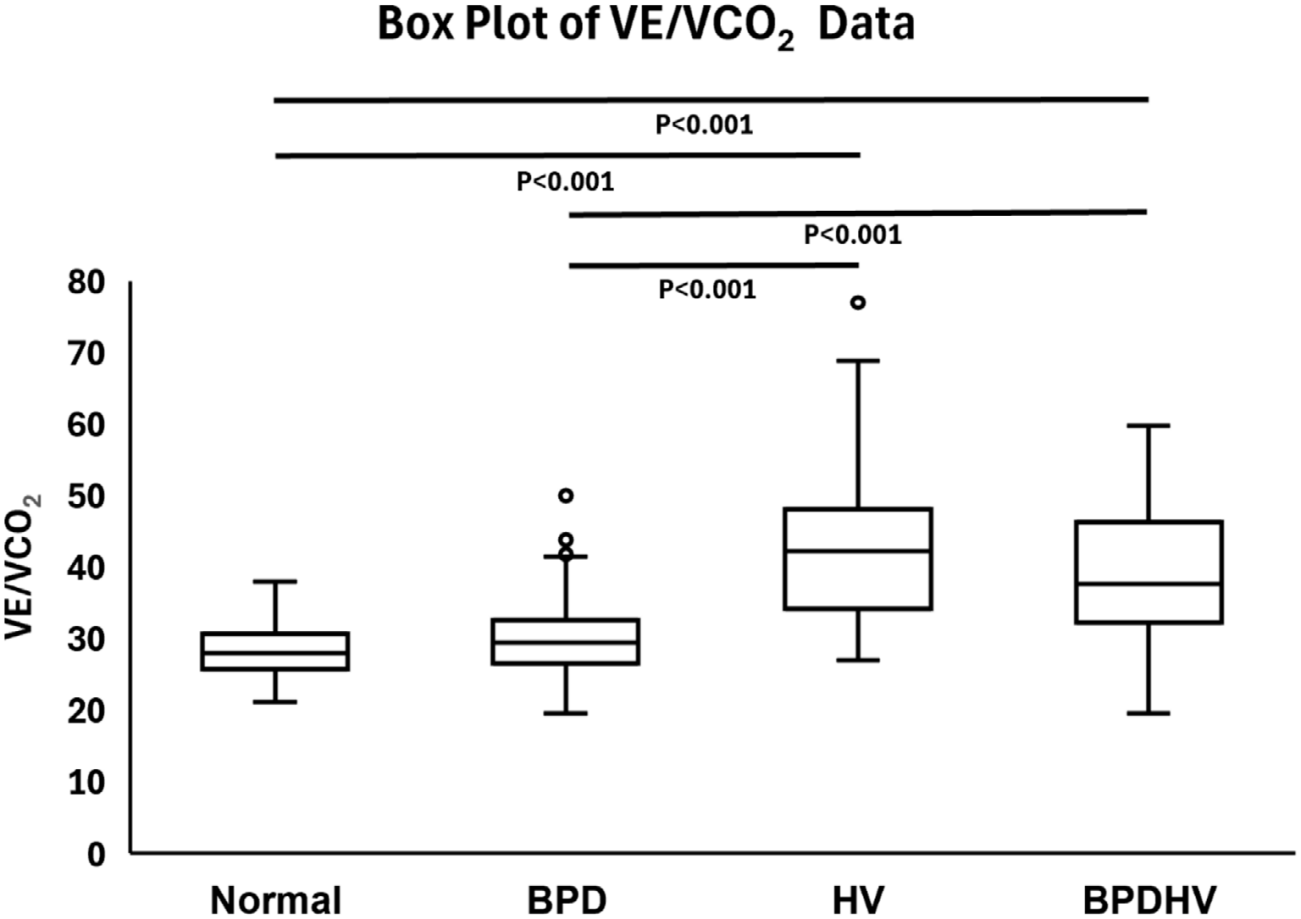
Box plot depicting VE/VCO_2_ data for each study group. The median, quartiles, minimum, maximum, and outliers are indicated. Significant pairwise comparisons as determined by post‐hoc DSCF tests are indicated with their associated *p*‐values.

VE/VCO_2_ differed significantly between study groups (Table 2). Patients with isolated BPD had similar ventilatory efficiency to normal (*p* = 0.09), while patients with HV or BPDHV had significantly impaired ventilatory efficiency (*p* < 0.001) (Figure 3).

### 3.4 Exercise performance

**FIGURE 4:**
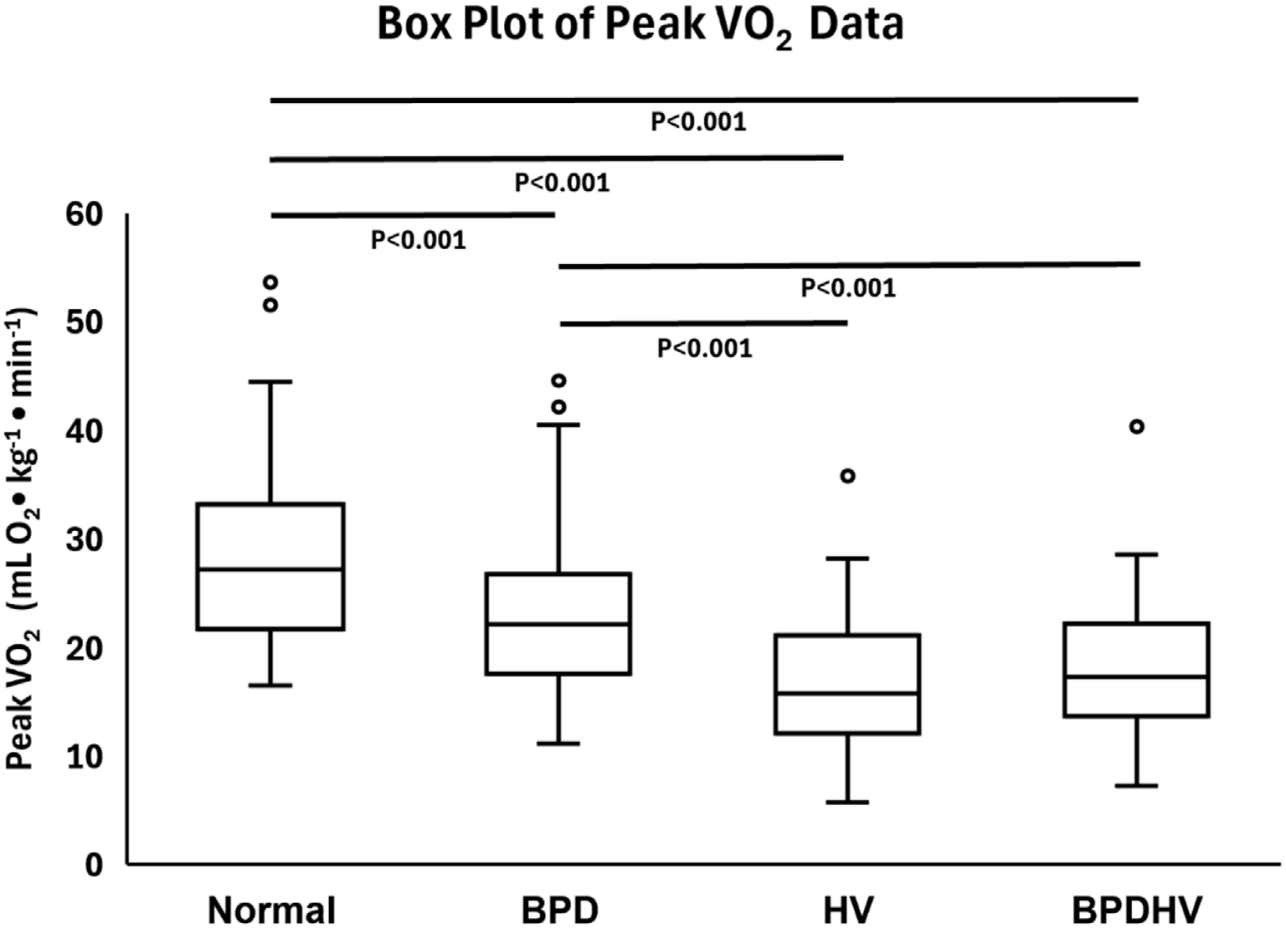
Box plot depicting Peak VO_2_ data for each study group. The median, quartiles, minimum, maximum, and outliers are indicated. Significant pairwise comparisons as determined by post‐hoc DSCF tests are indicated with their associated *p*‐values.

Median peak VO_2_ for each study group is presented in Table 2. Patients of all three dysfunctional breathing groups exhibited significantly impaired peak VO_2_ compared to those with normal CPETs (*p* < 0.001) (Figure 4). Patients with HV or BPDHV had significantly lower peak VO_2_ than patients with isolated BPD (*p* < 0.001) (Figure 4).

### 3.5 Functional impairment

**FIGURE 5:**
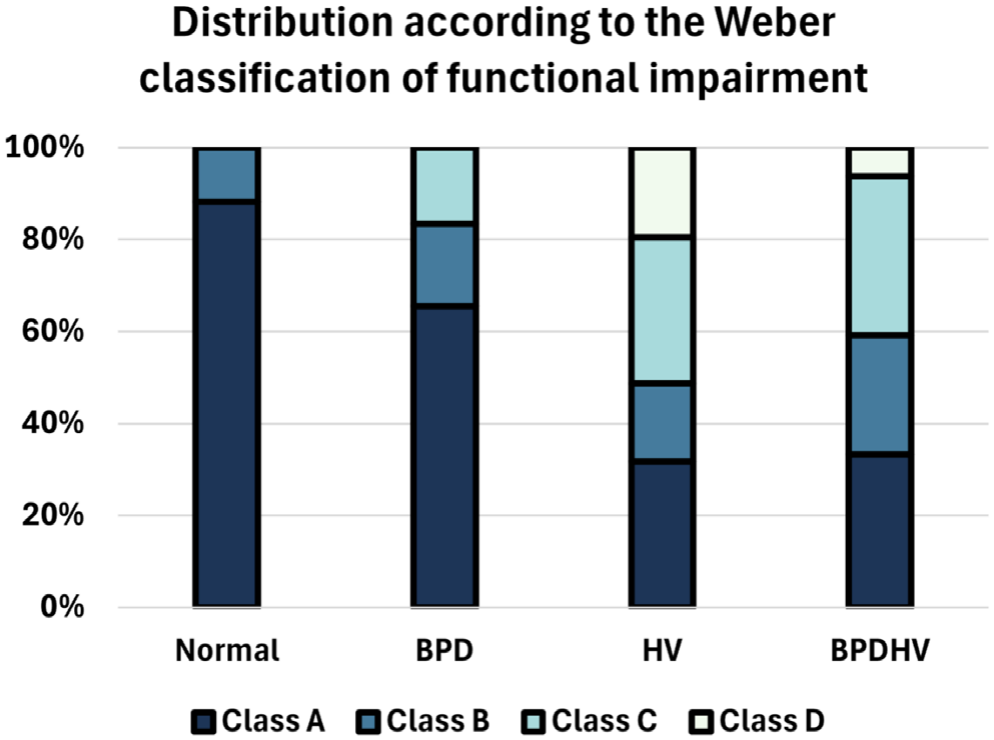
Stacked bar chart depicting the distribution of functional impairment for each study group according to the Weber classification. The relative percentage of patients in classes (a–d) for each study group are denoted by the different shades of blue.

The distribution of patients' functional capacity according to the Weber classification for each study group is presented in Figure 5. Patients with DB were more likely to be functionally impaired according to the Weber classification, with 35.5%, 68.3%, and 66.7% of patients Class B or above for BPD, HV, and BPDHV, respectively.

### 3.6 Relationship between VE/VCO_2_ and peak VO_2_

**FIGURE 6:**
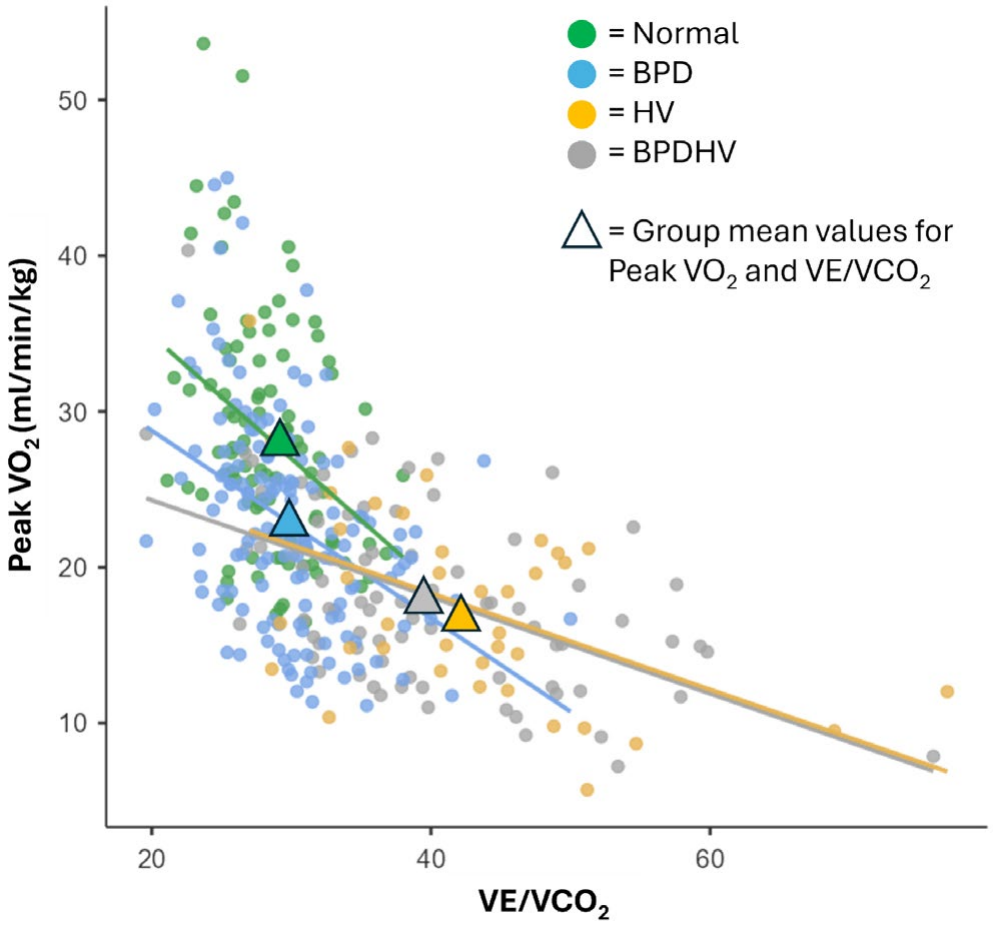
The relationship between VE/VCO_2_ and Peak VO_2_. Each study group is denoted as follows: 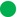 = Normal, 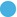 = BPD, 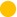 = HV, 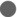 = BPDHV. Regression lines are also presented.

The relationship between VE/VCO_2_ and peak VO_2_ is illustrated in Figure 6. VE/VCO_2_ was found to correlate negatively with peak VO_2_ within each group (*p* < 0.01).

## 4 DISCUSSION

Dysfunctional breathing is a common driver of symptoms in patients referred for CPET due to unexplained dyspnoea, identified in approximately 40% in our cohort. Patients with BPD, HV, or BPDHV all had significantly impaired exercise capacity compared to the normal group and were more likely to be functionally impaired according to the Weber classification. VE/VCO_2_ was negatively correlated with peak VO_2_ across all groups.

DB was common in patients referred for CPET due to unexplained dyspnoea at our tertiary centre, identified as a driver of symptoms in 42.5%. Other studies investigating unexplained dyspnoea in specialized centres have reported prevalences of HV ranging from 8% to 20% (DePaso et al., 1991; Huang et al., 2017; Valentin et al., 2019), which are roughly in line with the overall 19.4% prevalence of BPDHV and HV in our study. However, studies rarely comment on the prevalence of BPD, highlighting this as an underrecognized driver of symptoms and a likely resultant underestimation of the prevalence of DB in patients with unexplained dyspnoea. DB is recognized to be a poorly understood condition by clinicians (Vidotto et al., 2019), with historical focus mainly on hyperventilation syndrome. Recently, breathing pattern disorder without hyperventilation has been highlighted as a driver of dyspnoea in ‘long COVID’ patients, affecting 27.5% of such patients in one cohort (Frésard et al., 2022). In recent studies, where the presence of both BPD and HV has been assessed, CPET DB prevalence in unexplained dyspnoea has been reported in the range 29.4%–62% (Frésard et al., 2022; Genecand et al., 2023; Knöpfel et al., 2024). Together with the present study, this emphasizes the high prevalence of DB—including both BPD and HV—in patients referred for CPET due to unexplained dyspnoea.

Significantly, more than half of patients (54.3%) identified with DB in the present study had isolated BPD. In current practice, BPD cannot be identified quantitatively, instead requiring subjective visual inspection of relevant data plots and pattern recognition to make the diagnosis (Ionescu et al., 2020). Indeed, both the normal group and isolated BPD group in our cohort had preserved ventilatory efficiency and did not differ significantly, with a median VE/VCO_2_ of 28.0 and 29.5, respectively (*p* > 0.05). Reported normal values for VE/VCO_2_ are generally suggested to be in the range 20–30 (Koch et al., 2009), with individualized upper limits of normal based on age, standing height, and weight from Gläser et al. (2013) suggesting a slight increase with age. VE/VCO_2_ therefore cannot be used as a quantitative measure to identify this subset of patients with DB. We would stress the importance of careful inspection of a combined plot detailing breathing frequency and tidal volume against minute ventilation in order to identify BPD, as detailed in Figure 1. This plot facilitates the identification of erratic or inappropriate changes of breathing frequency and/or tidal volume in response to increasing workload, characteristic of BPD. Visual plots of breathing patterns have the further advantage of guiding tailored therapeutic intervention, facilitating understanding and targeted breathing retraining of patient‐specific abnormal breathing responses to exercise.

Meanwhile, patients identified with HV and BPDHV in this study had significantly impaired ventilatory efficiency with a median VE/VCO_2_ of 42.3 and 37.7 respectively (*p* < 0.001). Such ventilatory inefficiency is characteristic of hyperventilation, in which the minute ventilation (VE) is excessive to exhale the CO_2_ produced (VCO_2_) at a given metabolic rate. A threshold of 35.0 at 40 W for the related parameter VeqCO_2_ (ventilatory equivalent for CO_2_) has previously been suggested as a diagnostic threshold for HV syndrome (Kinnula & Sovijärvi, 1993; Watson et al., 2021). In addition, end tidal pCO_2_ measurements <30 mmHg have also been proposed as a diagnostic threshold for HV syndrome (Ionescu et al., 2020). Increases in VE/VCO_2_ or VeqCO_2_ in isolation are not, however, specific to hyperventilation, with increases seen in a range of clinical conditions including heart failure, pulmonary hypertension, and chronic obstructive pulmonary disease (Phillips et al., 2020). A pilot study by Brat et al. (2019) sought to address this and showed that the absence of VeqCO_2_ and P_ET_CO_2_ changes during incremental exercise was highly specific for patients with HV versus controls. Future work is, however, required to verify these diagnostic thresholds in larger cohorts, and it is clear that a multi‐faceted approach is required to distinguish different causes of ventilatory inefficiency.

Patients of all three dysfunctional breathing groups exhibited significantly impaired peak VO_2_ compared to those with normal CPETs (*p* < 0.001). Patients with HV or BPDHV had significantly lower peak VO_2_ than patients with isolated BPD (*p* < 0.001), implying a role for hyperventilation as a driver of functional impairment, consistent with the reduced aerobic capacity identified in other cohorts of patients with hyperventilation syndrome (Brat et al., 2019; Chenivesse et al., 2014). When analyzed further, ventilatory efficiency, which is characteristically reduced in hyperventilation, was found to correlate negatively with peak VO_2_ within each group (*p* < 0.01) (Figure 6). VE/VCO_2_ may be a useful predictor of exercise impairment in patients with DB to guide therapeutic intervention. It is important to highlight, however, that patients with isolated BPD had significantly impaired peak VO_2_ independent of VE/VCO_2_. This is supported by data from Bansal et al. (2018), in which patients with BPD had a peak VO_2_ of 80% predicted, which was significantly lower than that of a control group, who had a mean peak VO_2_ of 124% predicted. The breathing pattern disorder itself, rather than ventilatory inefficiency, appears to drive functional impairment in these patients. Indeed, the approximate entropy of minute ventilation has been shown to be inversely related to peak VO_2_ (Bansal et al., 2018). In patients with symptom‐limited tests, dyspnoea was the most commonly cited reason for stopping, highlighting dysfunctional breathing‐induced sensations of breathlessness as a driver of functional limitation.

It has been noted that there is currently no way to quantify the severity of DB (Ionescu et al., 2020). The Weber classification of functional impairment is often used in heart failure to classify the severity of exercise impairment (Milani et al., 2004) and has been recommended for use in the interpretation of CPETs (Guazzi et al., 2012). In this study, we have applied the Weber classification to DB for the same purpose. Patients with DB were more likely to be functionally impaired (Class B or above), with some patients severely impaired in class D. This emphasizes the debilitating nature of DB, with functional impairment in some patients in line with that of severe heart failure. Indeed, several studies have revealed reduced quality of life in patients with DB (Chenivesse et al., 2014; Hagman et al., 2008). The Weber classification may therefore be a useful indicator of the functional impact of DB on patients, to help guide their management, and could be routinely included in CPET reports.

Demographic data was largely consistent across the four study groups of interest. Patients identified as having DB were typically in their 50s or 60s and clinically overweight (BMI 25–29.9 kg/m^2^). There was a slight female predominance in all study groups with no significant intergroup differences (*p* > 0.05). A significantly higher prevalence of DB in females has previously been reported in a primary care population (Thomas et al., 2005), suggesting DB may be more common in females. Patients with BPD and HV had a higher median BMI than patients with a normal CPET (*p* < 0.05). Interestingly, obesity is more commonly associated with obesity hypoventilation syndrome, in which hypoxic and hypercapnic ventilatory drive is reduced (Zwillich et al., 1975). It is plausible that DB induced exercise intolerance is a driver of obesity in these patients. This may further contribute to impaired exercise capacity through the effects of obesity on mechanical efficiency (Neder et al., 2018) and deconditioning associated with a sedentary lifestyle (Depiazzi & Everard, 2016).

## 5 LIMITATIONS AND FUTURE DIRECTIONS

This retrospective study did have some limitations. Given the retrospective nature of the study, it was not possible to identify a true “control” group. Instead, individuals in which no pathophysiological cause for their unexplained dyspnoea could be identified during CPET were included as a “Normal” group. This, however, could be considered a strength of the study, as all included patients had unexplained dyspnoea and in general were age matched. Patients with DB had significantly impaired peak VO_2_ when compared to this group; who themselves had reduced peak VO_2_ versus that of a typical sedentary male or female (Koch et al., 2009). Thus, the functional impairment induced by DB is likely even more severe than that identified in this study.

Some patients with DB did not attain a maximal CPET due to symptom limitation, as indicated by cardiac or ventilatory reserve preventing fulfillment of the criteria for a maximal CPET outlined by the ERS Standardization of CPETs statement (Radtke et al., 2019). Symptom limitation was most commonly due to feelings of dyspnoea, followed by leg fatigue and general exhaustion. Indeed, previous work by our team showed that individuals with HV were significantly less likely to obtain their VO_2_ max (Unpublished). It was therefore not possible to compare true VO_2_ max between groups. This, however, does not limit the value of the results; it is the symptom‐limited peak VO_2_ obtainable that reflects the functional limitation of patients with DB and the debilitating nature of the condition.

Pulmonary gas‐exchange impairment may also cause ventilatory inefficiency, with a consequent increased VE/VCO_2_ slope similar to that of hyperventilation. Pulmonary gas exchange can be assessed using a variety of non‐invasive measurements including O_2_ saturations, end tidal O_2_ or CO_2_, ventilatory slopes, and estimated physiologic dead space‐to‐tidal volume ratio (Vd/Vt) (American Thoracic Society and American College of Chest Physicians, 2003). Subtle gas exchange abnormalities may, however, require invasive measurement of arterial pO_2_ and determination of the alveolar–arterial PO_2_ pressure difference [P(a–a)O_2_]. Capillary blood gas measurements at rest and peak exercise are recommended during CPET for this purpose (Glaab & Taube, 2022). Some patients in our cohort did not, however, consent to blood gas measurements. It was therefore not possible to entirely rule out subtle gas exchange abnormalities in all patients; however, great care was taken to assess all available indicators of gas exchange in such cases, and a clinical diagnosis of DB was made if appropriate.

Another limitation of this study is the lack of standardized diagnostic criteria for BPD (Todd et al., 2018). Currently, there is no quantitative way to identify BPD in routine CPET interpretation (Knöpfel et al., 2024); CPET diagnosis of BPD relies on visual interpretation of relevant data plots in a 9‐panel plot by experienced clinicians (Ionescu et al., 2020). This study reveals a high prevalence of BPD in our cohort, which may be previously underdiagnosed by clinicians. It is therefore recommended that explicit inspection of the relevant plots for signs of BPD should be part of routine CPET interpretation. Future work is required, however, to formalize identification of BPD. Four pilot studies have attempted to quantify breathing pattern variability thus far via different methods: approximate entropy of minute ventilation across the entire CPET protocol (Bansal et al., 2018; Samaranayake et al., 2023), amplitude of changes of minute ventilation, breathing frequency or tidal volume at 3 stages of CPET (Mendes et al., 2023), and proportional tidal volume variation (Knöpfel et al., 2024). ROC curve analyses from these studies resulted in area under the curve (AUC) values ranging from 0.729 to 0.845, suggesting that quantification of breathing pattern variability and objective identification of BPD is a real possibility. Future work is required to compare and validate these methods in larger cohorts.

A further limitation of the present study was the pooling of different temporal patterns of DB. It is recognized that DB may present in different patterns in relation to exercise (Ionescu et al., 2020; Neder et al., 2018): DB may be present at rest, throughout incremental exercise, or at specific intensities of exercise. For the purposes of this study, temporal patterns of DB were not distinguished. This could have some ramifications in CPET interpretation; for example, select individuals with BPD or HV solely at rest or in low intensity exercise may subsequently have normal VE/VCO_2_ slopes, end tidal pCO_2_ values, and achieve their predicted peak VO_2_. This further reinforces the importance of visual interpretation of relevant plots and not solely relying on outputted values. This is unlikely to have affected the results in this study significantly, and if anything would reduce discrepancies between the DB and normal groups.

## 6 CONCLUSION

Dysfunctional breathing is common in patients referred for CPET due to unexplained dyspnoea, identified as a driver of symptoms in approximately 40% of this study cohort. More than half of these patients had isolated BPD, which requires visual inspection of relevant data plots to make the diagnosis. Those identified with dysfunctional breathing had significantly reduced functional exercise capacity, and the Weber classification of peak VO_2_ may be a useful classifier of functional severity in DB. While the diagnosis of DB is multi‐faceted, CPET is an invaluable tool in the identification and classification of different DB patterns—namely hyperventilation and/or breathing pattern disorder—with clinical importance in directing appropriate management and breathing retraining.

## FUNDING INFORMATION

This research received no external funding.

## CONFLICT OF INTEREST STATEMENT

The authors declare no conflict of interest.

## Data Availability

The anonymised data that support the findings of this study are not publicly available. The data are, however, available from the authors upon reasonable request, subject to permission being obtained from Cambridge University Hospitals.
